# Nothing but the truth? Effects of faking on the validity of the crosswise model

**DOI:** 10.1371/journal.pone.0258603

**Published:** 2021-10-29

**Authors:** Adrian Hoffmann, Julia Meisters, Jochen Musch

**Affiliations:** Department of Experimental Psychology, University of Duesseldorf, Duesseldorf, Germany; Sapienza, University of Rome, ITALY

## Abstract

In self-reports, socially desirable responding threatens the validity of prevalence estimates for sensitive personal attitudes and behaviors. Indirect questioning techniques such as the crosswise model attempt to control for the influence of social desirability bias. The crosswise model has repeatedly been found to provide more valid prevalence estimates than direct questions. We investigated whether crosswise model estimates are also less susceptible to deliberate faking than direct questions. To this end, we investigated the effect of “fake good” instructions on responses to direct and crosswise model questions. In a sample of 1,946 university students, 12-month prevalence estimates for a sensitive road traffic behavior were higher and thus presumably more valid in the crosswise model than in a direct question. Moreover, “fake good” instructions severely impaired the validity of the direct questioning estimates, whereas the crosswise model estimates were unaffected by deliberate faking. Participants also reported higher levels of perceived confidentiality and a lower perceived ease of faking in the crosswise model compared to direct questions. Our results corroborate previous studies finding the crosswise model to be an effective tool for counteracting the detrimental effects of positive self-presentation in surveys on sensitive issues.

## Introduction

When questioned about sensitive personal attributes, some individuals tend to answer in line with social norms rather than truthfully. Socially desirable responding results in an underestimation of socially undesirable attributes and an overestimation of socially desirable attributes [1–4]. To overcome social desirability bias, indirect questioning formats such as randomized response techniques [RRT; 5] have been proposed. RRTs grant full confidentiality to respondents by adding random noise to their answers. In the original RRT format, questions present participants with a sensitive statement A (e.g., “I have done cocaine”), and its negation, statement B (e.g., “I have never done cocaine”). Based on the outcome of a randomization procedure (e.g., the roll of a die), participants are instructed to respond to either statement A with probability *p* (e.g., “Respond to statement A if you rolled 1 or 2”; *p* = 2/6), or to statement B with probability 1-*p* (e.g., “Respond to statement B if you rolled any other number”; 1-*p* = 4/6). As the randomization outcome remains unknown to the experimenter, neither a “true” nor a “false” response reveals the respondent to be a carrier of the sensitive attribute. This is expected to increase the respondent’s motivation to answer truthfully. Moreover, since the probability *p* of selecting statement A is known, an estimate for the prevalence of the sensitive attribute can be obtained on the sample level. This estimate is assumed to be more valid than estimates obtained in conventional surveys, as it may be less biased by socially desirable responding [5].

Numerous studies have shown that conventional direct questions (DQ) do indeed lead to an underestimation of the prevalence of socially undesirable attributes. In contrast, RRT questions result in higher estimates that are considered to be less biased and hence more valid [e.g., 6,7–11]. Furthermore, meta-analyses have shown that RRT estimates often exceed DQ estimates, but still underestimate the true prevalence in studies in which the prevalence of the sensitive attribute is known [12]. Some studies even report RRT estimates comparable to, or lower than, DQ estimates [e.g., 13,14]. It is possible that such findings are due to respondents not understanding or not trusting the rather complex RRT procedure [15–17].

Consequently, nonrandomized response techniques [NRRT; 18], a recent advancement on RRT, promise to improve participants’ trust and understanding by providing simplified and more easily comprehensible instructions. The most frequently used NRRT is the crosswise model [CWM; 19]. Questions in the CWM format include two statements: a sensitive statement A (e.g., “I have done cocaine”), and a nonsensitive statement B with known prevalence (e.g., “I was born in November or December”; *p* = .158 according to official birth statistics provided by the German Federal Statistical Office). Participants are instructed to indicate whether “both statements are true, or both statements are false”, or “exactly one statement is true, irrespective of which one”. As in the original RRT, none of the answer options expose a respondent to be a carrier of the sensitive attribute. However, the prevalence of the sensitive attribute (π) can be estimated on the sample level using the formula:

$${\hat{\pi }}_{\mathrm{CWM}}=\frac{{\hat{\lambda }}_{\mathrm{CWM}}+p‐1}{2\;*\;p‐1}$$ (1)

where ${\hat{\lambda }}_{\mathrm{CWM}}$ is the observed proportion of respondents choosing the first answer option (“both statements are true, or both statements are false”).

The CWM has repeatedly been found to provide higher and, therefore, potentially more valid estimates for the prevalence of sensitive attributes than direct questions [e.g., 9,20–28]. Building on 45 empirical studies following this “more is better” validation approach, a recent meta-analysis supported the superiority of the CWM over DQ in controlling for the influence of socially desirable responses [29]. In addition, the CWM successfully recovered the true value in a “strong” validation study involving a sensitive attribute with known prevalence that served as an external validation criterion. In contrast, a direct question led to a vast underestimation [30]. However, more critical evaluations of the CWM have also suggested that the model is sometimes incapable of controlling for the influence of social desirability bias [e.g., 31–33]. More importantly, the CWM has been demonstrated to sometimes produce substantial shares of false positives (that is, non-carriers of the sensitive attribute falsely being classified as carriers) and false negatives (that is, carriers of the sensitive attribute falsely being classified as non-carriers) [34,35]. Both false positives and false negatives are likely attributable to respondents failing to understand the CWM instructions and choosing their answer at random. Such answering behavior potentially distorts prevalence estimates towards 50%, and is therefore especially problematic when socially undesirable attributes with a zero, or very low, prevalence are investigated. In these cases, a substantial share of random responses can inflate CWM estimates and thereby lead to problematic overestimations [36,37]. A recent investigation of the CWM has, however, shown that for undesirable attributes with a prevalence well above 0%, the inflating effect of false positives was outweighed by a stronger deflating influence of false negatives, resulting in an overall under- rather than an overestimation of the true prevalence [38]. Taken together, the current empirical and meta-analytic evidence suggests that CWM prevalence estimates for socially undesirable attributes are not necessarily perfect, but likely closer to the true value than DQ estimates, and therefore usually more valid.

The overall mostly positive results are presumably due to the comparatively high comprehensibility of CWM instructions. Unlike the original RRT, the CWM integrates the required randomization directly into the answer options, thereby eliminating the need for an external randomization device. This arguably makes the method easier to administer for both the interviewer and the interviewee [19]. Supporting this assumption, the CWM has been evaluated as the most comprehensible format among several indirect questioning techniques and exhibits a significant improvement on perceived privacy protection compared to direct questions [39].

Most importantly, however, the CWM offers symmetrical answer options. None of the available answers represents a “safe” alternative respondents can choose in order to explicitly deny being a carrier of the sensitive attribute. This property of the model can be demonstrated by computing the conditional probabilities [40,41] of being identified as a carrier of the sensitive attribute when choosing the first (“both statements are true, or both statements are false”) versus the second answer option (“exactly one statement is true, irrespective of which one”) using Bayes’ formula [42]:

$${\mathrm{Pr}}_{\mathrm{CWM}}\left(\mathrm{carrier}|“\mathrm{both}/\mathrm{none}\;\mathrm{true}”\right)=\frac{{\mathrm{Pr}}_{\mathrm{CWM}}(\mathrm{carrier}\cap “\mathrm{both}/\mathrm{none}\;\mathrm{true}”)}{{\mathrm{Pr}}_{\mathrm{CWM}}(“\mathrm{both}/\mathrm{none}\;\mathrm{true}”)}$$ (2.1)

$${\mathrm{Pr}}_{\mathrm{CWM}}\left(\mathrm{carrier}|“\mathrm{one}\;\mathrm{true}”\right)=\frac{{\mathrm{Pr}}_{\mathrm{CWM}}(\mathrm{carrier}\cap “\mathrm{one}\;\mathrm{true}”)}{{\mathrm{Pr}}_{\mathrm{CWM}}(“\mathrm{one}\;\mathrm{true}”)}$$ (2.2)

These equations can be reformulated using the parameters for prevalence estimation from Eq 1:

$${\mathrm{Pr}}_{\mathrm{CWM}}(\mathrm{carrier}|“\mathrm{both}/\mathrm{none}\;\mathrm{true}”)=\frac{{\hat{\pi }}_{\mathrm{CWM}}\;*\;p}{{\hat{\lambda }}_{\mathrm{CWM}}}$$ (2.3)

$${\mathrm{Pr}}_{\mathrm{CWM}}\left(\mathrm{carrier}|“\mathrm{one}\;\mathrm{true}”\right)=\frac{{\hat{\pi }}_{\mathrm{CWM}}\;*\;(1‐p)}{\left(1‐{\hat{\lambda }}_{\mathrm{CWM}}\right)}$$ (2.4)

As can be seen in Eqs 2.3 and 2.4, the probability of being identified as a carrier of the sensitive attribute exceeds zero regardless of whether respondents choose the first or the second answer option for all cases of 0 < ${\hat{\pi }}_{\mathrm{CWM}}$ < 1, 0 < *p* < 1, and 0 < ${\hat{\lambda }}_{\mathrm{CWM}}$ < 1. These conditions are usually met in applications of the CWM, because researchers typically ensure that the expected prevalence of the sensitive attribute, the randomization probability, and the proportion of respondents choosing the first answer option are different from 0% and 100%. Under such conditions, no “safe” answer option is available for respondents to choose in order to explicitly deny being a carrier of the sensitive attribute. Despite the absence of an objectively safe answer, respondents confronted with a CWM question might nevertheless try to assess the risk of being classified as a carrier of the sensitive attribute as a function of the answer option they decide to select. As neither the prevalence of the sensitive attribute (${\hat{\pi }}_{\mathrm{CWM}}$) nor the proportion of respondents choosing the first answer option (${\hat{\lambda }}_{\mathrm{CWM}}$) is known until data collection is complete, it is impossible for respondents to calculate the exact conditional probabilities depicted in Eqs 2.3 and 2.4, respectively. As can, however, also be seen when comparing the numerators of Eqs 2.3 and 2.4, the relative risk of being identified as a carrier is lower when choosing the first (“both/none true”) rather than the second answer option (“one true”) in all cases of 0 < *p* < .5, because *p* < 1 − *p*. For .5 < *p* < 1, the second answer option is associated with a lower risk because *p* > 1 − *p*. Consequently, participants following a self-protective response strategy could try to compare the relative risk of the two answer options based on the randomization probability, and then choose the less risky option. To do so, they would have to (a) correctly estimate the randomization probability *p*, and (b) derive and understand the relationship between the randomization probability and the conditional probabilities of being identified as a carrier when choosing either of the two answer options. Previous studies have shown that most respondents are poor at estimating the relationship between the randomization probability and the objective privacy protection afforded by indirect questioning techniques, except for cases in which extreme randomization probabilities eliminate confidentiality [43]. In addition, we believe that the necessary logical deductions and calculations are too time-consuming for common short survey situations. We therefore argue that it is improbable that a substantial share of respondents confronted with a CWM question will succeed in identifying a self-protective response. Because response symmetry also reduces the incentive to provide untruthful answers [44] and because the symmetrical CWM has been shown to provide potentially more valid estimates than a related model with asymmetric response options [26], we propose that the symmetry of the CWM will lead to a higher proportion of honest responses compared to a direct question, even or especially if respondents are incentivized to provide self-protective responses by asking them to make a positive impression. Our expectation was that respondents trying to follow the instruction to provide self-protective responses would be able to successfully change their answers to a direct question in order to make a positive impression, but would be much less successful when answering indirect CWM questions. We therefore surmised that “fake good” instructions would severely impair the validity of direct questioning estimates, whereas crosswise model estimates would be much less vulnerable to a deliberate faking attempt.

As a sensitive attribute, we chose to ask participants whether they had crossed the street on a “Don’t Walk” sign in plain sight of children within the past twelve months. Crossing the street on a “Don’t Walk” sign is a common behavior among German and Austrian adults, but is exhibited less frequently when children are present [45,46]. This is presumably because adults are aware of their function as a role model in street-crossing behavior for their own as well as other people’s children [46]. Nevertheless, a considerable portion of the population still crosses the street on a “Don’t Walk” sign even when in plain sight of children [46–48]. We therefore expected that a substantial share of the participants in our sample had exhibited this behavior in the past twelve months, but would feel reluctant to admit it due to self-presentation concerns.

Previous studies on the CWM have tended to investigate the validity of its prevalence estimates or variables that can elicit truthful responses, such as trust or understanding of the method. The present study extends these findings by conducting the first experimental examination of the influence of deliberate positive self-presentation. To this end, we obtained DQ and CWM prevalence estimates for a sensitive attribute in an “honest” control condition, in which participants were instructed to respond truthfully, and compared these estimates to those obtained in an experimental “fake good” condition, in which participants were instructed to respond in a way that would leave a positive impression. Similar “fake good” manipulations have successfully been employed to investigate the influence of positive self-presentation on, for example, the validity of personality tests [49–52], social desirability scales [53], gender differences in self-presentation [54], and prejudice [55]. Our decision to use an experimental “fake good” manipulation pursued the goal of maximizing experimental control. We had to accept that our decision to prioritize internal validity came at the cost of decreasing ecological validity compared to real-world situations differing in their incentive to fake. However, any non-experimental approach would have provided less internal validity. We expected that direct self-reports in the DQ condition would be prone to the influence of social desirability, resulting in a substantial underestimation of the prevalence of crossing the street on a “Don’t Walk” sign in plain sight of children. We expected CWM estimates to be higher than DQ estimates to the extent to which indirect questioning is capable of controlling for socially desirable responding. Applying the “more is better”-criterion, these higher estimates can be considered more valid and presumably less biased [56]. We also expected that deliberate attempts to fake good would severely impair the validity of DQ estimates, leading to considerably lower and therefore presumably less valid estimates in the “fake good” condition. Finally, if the CWM is indeed robust against deliberate faking due to respondents’ inability to identify a self-protective answer, a much smaller (if any) difference between the “honest” and “fake good” conditions can be expected for CWM estimates.

In summary, the present study is the first to investigate the influence of a “fake good” manipulation on the validity of prevalence estimates obtained via indirect questioning techniques. As expected, we could show that deliberate positive self-presentation severely impaired the validity of prevalence estimates obtained via a conventional direct question, while estimates based on the CWM were largely unaffected.

## Methods

### Participants

A total of 2,024 subjects participated in our survey. Due to incomplete information on the questionnaire, 78 participants (3.9%) had to be excluded from further analyses. The final sample thus consisted of *N* = 1,946 respondents, of which 52.4% were female. The mean age was 20.9 years (*SD* = 4.58). Participants were recruited and assessed in lecture halls and public spaces at the Universities of Duesseldorf (71.2%), Aachen (21.2%) and Cologne (7.7%), Germany. The survey was carried out in accordance with the revised Declaration of Helsinki [57] and the ethical guidelines of the German Association of Psychologists and the German Psychological Society [58]. Written information on the survey content, the strict anonymization of all personal data, and the exclusive use of the collected data for research purposes was provided directly on the questionnaire. Potential participants were instructed to document their consent by filling out and returning the questionnaire to the experimenter; they were explicitly asked not to return the questionnaire should they not consent to participate. Participation was voluntary and not associated with any risk of physical or mental harm or discomfort beyond participants’ everyday experiences. Therefore, ethics committee approval was not required according to the “Ethical Research Principles and Test Methods in the Social and Economic Sciences” formulated by the Ethics Research Working Group of the German Data Forum [59] and the “Ethical recommendations of the German Psychological Society for researchers and ethics committees” [60]. A small proportion of participants (1.7%) were underage because in Germany, some pupils finish high school and start attending university prior to their 18th birthday. For these students, parents had provided written consent for them to partake in all study-related activities, including participation in the present study.

The prevalence of the sensitive attribute at the population level, as well as the prevalence estimates in each subgroup, were unknown before the study was conducted. Therefore, a priori power considerations regarding the required sample size were initially made so that an acceptable power (1-β > .80) for detecting any proportion of carriers of the sensitive attribute in the direct and indirect questioning conditions ($\hat{\pi }$ > 0%) would likely be achieved. These considerations revealed that a total sample size of *N* ≥ 1,500, and an allocation of twice as many participants to the indirect compared to the direct questioning groups would ensure sufficient statistical power for the planned prevalence estimations [cf. 61].

For differences between experimental groups concerning the three additional variables–perceived sensitivity of the topic, perceived confidentiality, and subjective ease of faking–, we performed sensitivity analyses, as well as post-hoc power analyses, using the software G*Power 3 [62]. Based on our final sample size and a desired Type-I error probability of α = .05, sensitivity analyses revealed that for all main and interaction effects in the ANOVAs, sufficient statistical power (1-β > .80) was achieved for a minimum effect size of *f* ≥ .06, and thus even for small effects; post-hoc power analyses further confirmed that the achieved power was very high (1-β > .99) for large (*f* ≥ .40), medium (*f* ≥ .25), and small effects (*f* ≥ .10). Sensitivity analyses, and post-hoc power analyses, for pairwise t-tests were performed applying Bonferroni corrections for multiple testing. As for each of the three additional variables, four pairwise comparisons were conducted, Bonferroni correction resulted in an adjusted Type-I error level of α_adj_ = α / 4 = .0125. Given the distribution of participants across experimental groups, sensitivity analyses revealed that the minimum effect size for which sufficient statistical power (1-β > .80) was achieved was small, and ranged from *d* ≥ 0.19 for the comparison of “CWM, honest” to “CWM, fake good” conditions, to *d* ≥ 0.26 for the comparison of “DQ, honest” to “DQ, fake good” conditions. Post-hoc power analyses further showed that the achieved power was very high (1-β > .99) for all pairwise comparisons when assuming large (*d* ≥ .80) or medium effects (*d* ≥ .50). For small effects (*d* ≥ .20), sufficient power was achieved for the comparison of “CWM, honest” to “CWM, fake good” conditions (1-β = .86); however, power was less optimal for the comparisons of “DQ, honest” to “CWM, honest” conditions (1-β = .68), of “DQ, fake good” to “CWM, fake good” conditions (1-β = .67), and of “DQ, honest” to “DQ, fake good” conditions (1-β = .53).

### Survey design

All instructions and questions were placed on a single-page paper-pencil questionnaire. The introduction section informed participants that they would be asked questions about serving as a role model for children in everyday traffic situations. In the experimental part of the questionnaire, a black-rimmed box provided “honest” versus “fake good” instructions and posed the experimental question in either a direct (DQ) or crosswise model (CWM) format. At the bottom of the questionnaire, five additional questions assessed participants’ demographics and experience of the survey. The 2x2 between-subjects design resulted in four versions of the questionnaire, which differed only with respect to the content of the central black-rimmed box. Depending on condition, this box contained (a) the instruction to respond honestly to the question in DQ format; (b) the instruction to respond honestly to the question in CWM format; (c) the instruction to fake good when answering the question in DQ format, or (d) the instruction to fake good when answering the question in CWM format. To compensate for the lower efficiency of indirect questioning techniques resulting from the required randomization, twice as many participants were assigned to the CWM conditions (b) and (d) than to the DQ conditions (a) and (c) [cf. 61,63]. The distribution of participants across experimental conditions is shown in Table 1. The original German questionnaire used for data collection along with an English translation is available on the Open Science Framework (https://doi.org/10.17605/OSF.IO/KJZQR).

**Table 1:** Distribution of participants across experimental conditions.

		Questioning technique				Total	
		DQ		CWM			
						
Instruction	“honest”	*n*_(a)_ = 334 (17.16%)		*n*_(b)_ = 637 (32.73%)		971 (49.90%)	
	“fake good”	*n*_(c)_ = 326 (16.75%)		*n*_(d)_ = 649 (33.35%)		975 (50.10%)	
							
Total		660 (33.92%)		1286 (66.08%)			


DQ = direct questioning, CWM = crosswise model.

#### Instructions to respond honestly versus fake good

In the honest conditions (a) and (b), the following text was printed in bold red letters to attract participants’ attention and placed right before the experimental question: “We are interested in the incidence of certain behaviors in traffic situations. Hence, please respond honestly to the question in this black-skimmed box, and report your actual previous behavior.” In the fake good conditions (c) and (d), this passage read: “We are interested in how dishonest responding affects survey results. Hence, please do not respond honestly to the question in this black-skimmed box, but in a way that will leave as positive an impression of yourself as possible.”

#### Questioning technique

In the DQ conditions (a) and (c), we presented a single sensitive statement for which the prevalence π_DQ_ was to be estimated: “Within the past 12 months, I have crossed the street on a ‘Don’t Walk’ sign even though I was in plain sight of a child.” Participants were asked to indicate whether this statement was “true” or “false”. In the CWM conditions (b) and (d), we presented two statements simultaneously – a sensitive statement A for which the prevalence π_CWM_ was to be estimated, and a nonsensitive statement B with known prevalence *p* (Statement A: “Within the past 12 months, I have crossed the street on a ‘Don’t Walk’ sign even though I was in plain sight of a child.”; Statement B: “I was born in November or December.”). The prevalence for statement B was known to be *p* = .158 according to official birth statistics. Participants had to choose between the two answer options “I agree with both statements or with none of the statements”, and “I agree with exactly one statement (irrespective of which one)”.

#### Additional variables

To further explore the participants’ experience, we included three additional questions asking about the perceived sensitivity of the question topic, the perceived confidentiality offered by the questioning technique used in the respective condition, and the subjective ease of faking on the questionnaire.

To assess the perceived sensitivity of the topic, we asked: “How bad do you think it is when an adult crosses the street on a ‘Don’t Walk’ sign in plain sight of children?” Responses were recorded on a 7-point Likert scale ranging from “not bad at all” (1) to “very bad” (7). To measure the perceived confidentiality offered by the questioning technique, we presented the question: “How well do you think the confidentiality of your answer is protected in the above question?” Subjects answered on a 7-point Likert scale ranging from “confidentiality is not granted at all” (1) to “confidentiality is granted in an optimal way” (7). To assess the subjective ease of faking on the questionnaire, we asked: “How easy do you think it is to answer the above question in such a way that you give the impression that you have never crossed the street on a ‘Don’t Walk’ sign in plain sight of children?” Subjects were required to indicate their response on a 7-point Likert scale ranging from “very easy” (1) to “very hard” (7).

### Statistical analyses

**Fig 1:**
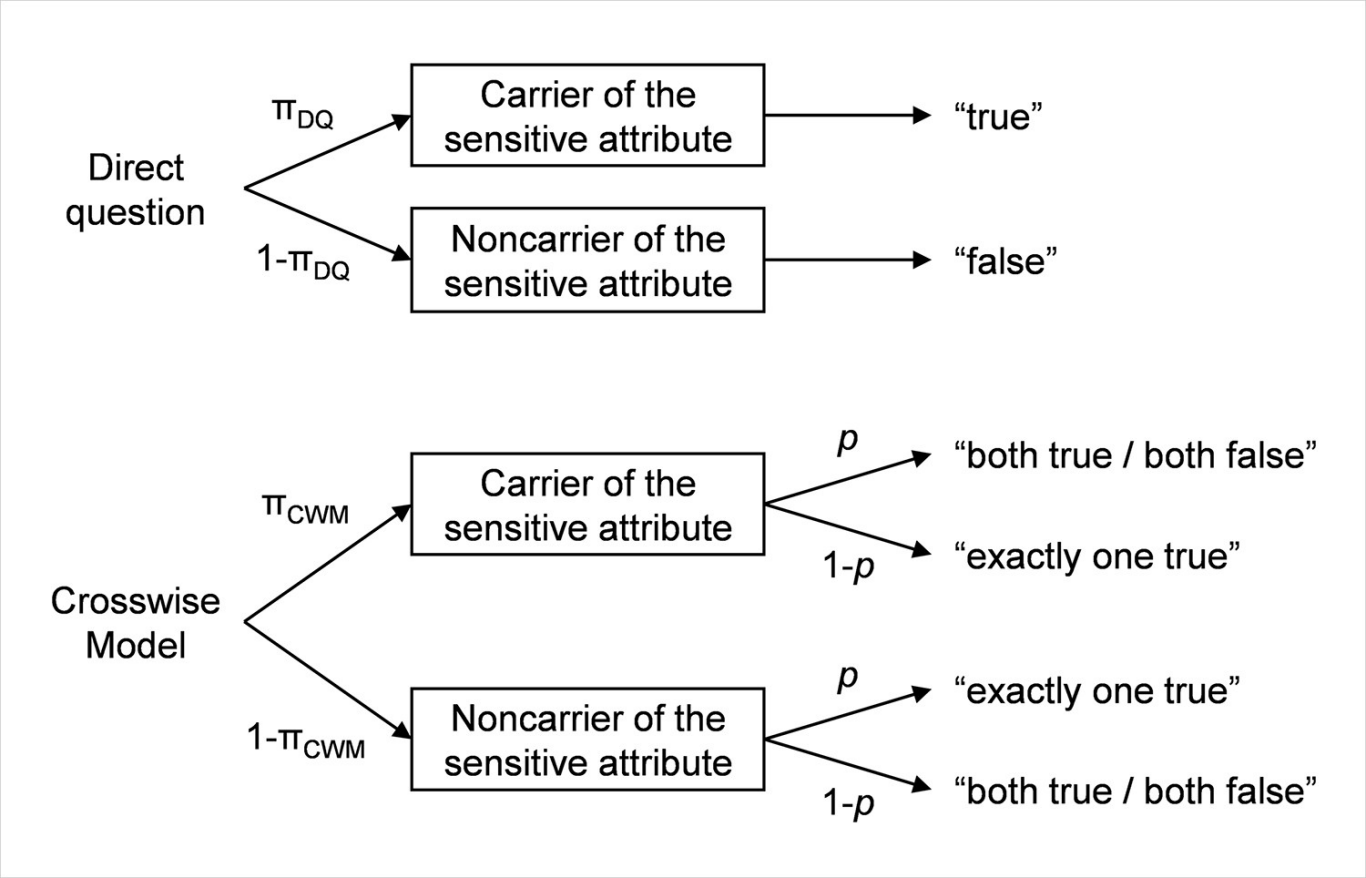
Tree diagram of the multinomial processing trees established for the direct questioning and the crosswise model conditions, respectively. π = unknown prevalence of the sensitive attribute, *p* = known randomization probability.

To estimate the prevalence of the sensitive attribute, we used multinomial processing tree models [64,65] following the procedure detailed in, for example, [7,63,66]. To evaluate the influence of the independent variables *instruction* (“honest” versus “fake good”) and *questioning technique* (“DQ” versus “CWM”), separate multinomial processing trees were formulated for each experimental condition (a) to (d). Within each processing tree, a parameter π represented the prevalence estimate of the sensitive attribute. In the CWM conditions, an additional parameter *p* reflected the probability of being born in November or December, which was used for randomization. This probability was known to be 15.8% from official birth statistics provided by the German Federal Statistical Office [67], and was thus set constant to *p* = .158. As an example, the processing trees established for the direct questioning versus crosswise model conditions are shown in Fig 1.

We computed maximum likelihood estimates for π on the basis of the empirically observed answer frequencies using the expectation maximization algorithm [68,69] as implemented in the software multiTree [70]. To assess differences between parameter estimates (for example, ${\hat{\pi }}_{\mathrm{DQ},\;“\mathrm{honest}”}$ versus ${\hat{\pi }}_{\mathrm{CWM},\;“\mathrm{honest}”}$), we compared unrestricted baseline models in which both parameters could be estimated freely to restricted alternative models in which both parameters were set to be equal (for example, π_DQ, “honest”_ = π_CWM, “honest”_). In these model comparisons, significant changes in the asymptotically Χ^2^-distributed log-likelihood statistic *G^2^* indicate that the restricted alternative model fits the data worse than the baseline model. If this is the case, the parameter restriction in the alternative model is shown to be inadmissible, and the two parameter estimates are shown to differ significantly (for example, ${\hat{\pi }}_{\mathrm{DQ},\;“\mathrm{honest}”}$ ≠ ${\hat{\pi }}_{\mathrm{CWM},\;“\mathrm{honest}”}$).

To investigate a potential interaction between instruction and questioning technique, we introduced parametric order constraints [71] by reparameterizing the original model as detailed in Hoffmann and Musch (28). In the reparameterized model, the shrinkage parameter α_DQ_ represented the ratio of the estimated prevalence in the “DQ, ‘fake good’” condition (π_DQ, “fake good”_) to the estimated prevalence in the “DQ, ‘honest’” condition (π_DQ, “honest”_); likewise, parameter α_CWM_ represented the ratio of the estimated prevalence in the “CWM, ‘fake good’” condition (π_CWM, “fake good”_) to the estimated prevalence in the “CWM, ‘honest’” condition (π_CWM, “honest”_). A significant difference between the estimated shrinkage ratios ${\hat{\alpha }}_{\mathrm{DQ}}$ and ${\hat{\alpha }}_{\mathrm{CWM}}$, assessed via a *G^2^* test as described above, indicated a significant interaction between instruction and questioning technique.

The effects of instruction (“honest” versus “fake good”) and questioning technique (DQ versus CWM) on the three additional variables perceived sensitivity of the topic, perceived confidentiality, and subjective ease of faking were assessed via three 2x2 between-subjects ANOVAs. Pairwise comparisons between specific experimental groups (for example, “DQ, ‘honest’” versus “DQ, ‘fake good’”) were conducted via t-tests for independent samples; *p*-values for these pairwise comparisons were Bonferroni-corrected to account for multiple testing.

A raw dataset containing respondents’ answers to the experimental and additional questions, as well as all multinomial model equations and empirically observed answer frequencies necessary to reproduce the parameter estimates reported in this manuscript, are available on the Open Science Framework (https://doi.org/10.17605/OSF.IO/KJZQR).

## Results

### Main results: Prevalence of the sensitive attribute

Parameter estimates and parameter comparisons for the sensitive attribute are shown in Table 2. As expected, the prevalence estimate for the sensitive attribute was substantially higher, and thus presumably more valid, in the conditions with a crosswise model question (CWM) rather than direct questioning (DQ). This was true for both “honest” and “fake good” instructions. Furthermore, “fake good” instructions resulted in substantially lower prevalence estimates than “honest” instructions in the DQ condition, but not in the CWM condition.

**Table 2:** Parameter estimates (standard errors in parentheses) and parameter comparisons for the prevalence of the sensitive attribute (“Within the past 12 months, I have crossed the street on a ‘Don’t Walk’ sign even though I was in plain sight of a child.”).

Parameter estimates		Questioning technique		
		DQ	CWM	
*Estimated prevalence* $\hat{\pi }$				
Instruction	“honest”	35.03% (2.61)	45.53% (2.89)	
	“fake good”	15.64% (2.01)	42.00% (2.85)	
*Estimated shrinkage* $\hat{\alpha }$				
“Fake good” relative to “honest” condition		44.66% (6.64)	92.26% (8.58)	
Parameter comparisons			Model fit	
		|*difference*|	Δ*G^2^* (*df* = 1)	*p*
*Instruction*				
	${\hat{\pi }}_{\mathrm{DQ},\;“\mathrm{honest}”}\;=\;{\hat{\pi }}_{\mathrm{DQ},\;“\mathrm{fake}\;\mathrm{good}”}$	19.39%	33.40	<.001 *
	${\hat{\pi }}_{\mathrm{CWM},\;“\mathrm{honest}”}\;=\;{\hat{\pi }}_{\mathrm{CWM},\;“\mathrm{fake}\;\mathrm{good}”}$	3.53%	0.75	.386
*Questioning technique*				
	${\hat{\pi }}_{\mathrm{DQ},\;“\mathrm{honest}”}\;=\;{\hat{\pi }}_{\mathrm{CWM},\;“\mathrm{honest}”}$	10.50%	7.18	.007 *
	${\hat{\pi }}_{\mathrm{DQ},\;“\mathrm{fake}\;\mathrm{good}”}\;=\;{\hat{\pi }}_{\mathrm{CWM},\;“\mathrm{fake}\;\mathrm{good}”}$	26.36%	52.97	< .001 *
*Instruction* * *Questioning technique*				
	${\hat{\alpha }}_{\mathrm{DQ}}\;=\;{\hat{\alpha }}_{\mathrm{CWM}}$	47.60%	18.12	< .001 *

* significant at *p* < .05.

A significant difference between the shrinkage ratios ${\hat{\alpha }}_{\mathrm{DQ}}$ and ${\hat{\alpha }}_{\mathrm{CWM}}$ indicated a significant interaction between questioning technique and instruction (Δ*G^2^* [*df* = 1] = 18.12, *p* < .001). A shrinkage ratio of ${\hat{\alpha }}_{\mathrm{DQ}}$ = 45% revealed that in the DQ condition, the estimated prevalence ${\hat{\pi }}_{\mathrm{DQ},\;“\mathrm{fake}\;\mathrm{good}”}$ was only .45 times the size of the estimated prevalence ${\hat{\pi }}_{\mathrm{DQ},\;“\mathrm{honest}”}$; thus, participants were less than half as likely to admit to the sensitive attribute when instructed to fake good compared to when instructed to respond honestly. In the CWM condition, a shrinkage ratio of ${\hat{\alpha }}_{\mathrm{CWM}}$ = 92% suggested that the estimated prevalence ${\hat{\pi }}_{\mathrm{CWM},\;“\mathrm{fake}\;\mathrm{good}”}$ was .92 times the size of, and therefore roughly comparable to, the estimated prevalence ${\hat{\pi }}_{\mathrm{CWM},\;“\mathrm{honest}”}$. Hence, fake good instructions severely impaired the validity of prevalence estimates obtained via DQ, but hardly affected the validity of estimates obtained via the CWM.

### Exploratory analyses: Additional variables on subjective experience

**Fig 2:**
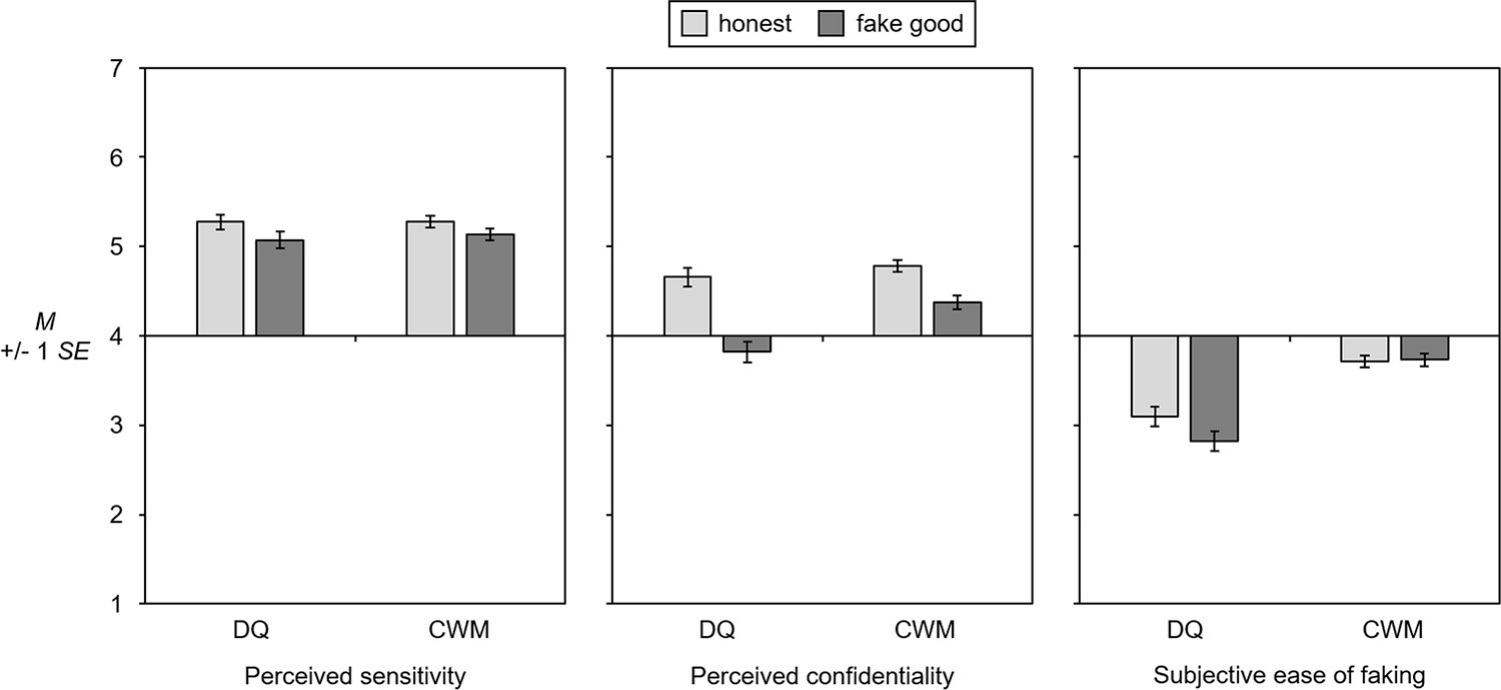
Mean plots for perceived sensitivity (higher values = higher sensitivity), perceived confidentiality (higher values = higher confidentiality), and subjective ease of faking (higher values = harder to fake). DQ = direct questioning, CWM = crosswise model.

Results for the additional variables perceived sensitivity of the topic, perceived confidentiality, and subjective ease of faking are shown in Table 3; plots of the observed means are shown in Fig 2.

**Table 3:** Descriptive statistics, ANOVA results, and pairwise t-test results for perceived sensitivity, perceived confidentiality, and subjective ease of faking.

Descriptive statistics		Questioning technique	
		DQ	CWM
		*M (SE)*	*M (SE)*
*Perceived sensitivity of the topic (higher values = higher perceived sensitivity)*			
Instruction	“honest”	5.27 (0.08)	5.28 (0.07)
	“fake good”	5.07 (0.09)	5.14 (0.07)
*Perceived confidentiality (higher values = higher perceived confidentiality)*			
Instruction	“honest”	4.66 (0.10)	4.78 (0.07)
	“fake good”	3.82 (0.12)	4.37 (0.08)
*Subjective ease of faking (higher values = subjectively harder to fake)*			
Instruction	“honest”	3.10 (0.11)	3.71 (0.07)
	“fake good”	2.83 (0.11)	3.73 (0.07)


ANOVAs	*F* (1, 1942)	*p*	*f*
			
*Perceived sensitivity of the topic*			
Instruction (“honest” vs. “fake good”)	4.73	.030 *	.049
Questioning technique (DQ vs. CWM)	0.20	.653	.010
Instruction * Questioning technique	0.13	.719	.008
*Perceived confidentiality*			
Instruction (“honest” vs. “fake good”)	47.81	< .001 *	.157
Questioning technique (DQ vs. CWM)	14.32	< .001 *	.086
Instruction * Questioning technique	5.75	.017 *	.054
*Subjective ease of faking*			
Instruction (“honest” vs. “fake good”)	1.94	.164	.032
Questioning technique (DQ vs. CWM)	73.34	< .001 *	.194
Instruction * Questioning technique	2.70	.101	.037

Pairwise t-tests		*t*	*df*	*p* ^†^	*d*
*Perceived sensitivity of the topic*					
DQ	“honest” vs. “fake good”	1.61	658	.430	0.126
CWM	“honest” vs. “fake good”	1.53	1284	.502	0.084
“Honest”	DQ vs. CWM	0.06	969	> .999	0.006
“Fake good”	DQ vs. CWM	0.57	973	> .999	0.042
*Perceived confidentiality*					
DQ	“honest” vs. “fake good”	5.42	658	< .001 *	0.424
CWM	“honest” vs. “fake good”	4.00	1284	< .001 *	0.226
“Honest”	DQ vs. CWM	1.03	969	> .999	0.066
“Fake good”	DQ vs. CWM	4.17	973	< .001 *	0.276
*Subjective ease of faking*					
DQ	“honest” vs. “fake good”	1.75	658	.323	0.136
CWM	“honest” vs. “fake good”	-0.22	1284	> .999	0.011
“Honest”	DQ vs. CWM	5.02	969	< .001 *	0.329
“Fake good”	DQ vs. CWM	7.04	973	<.001 *	0.466

* significant at *p* < .05.

^†^ Bonferroni-corrected for multiple testing (original *p* values multiplied by 4).

Analyses of the perceived sensitivity question revealed that participants considered crossing the street on a ‘Don’t Walk’ sign in plain sight of children to be rather poor behavior, as reflected in mean values of 5.07 to 5.28 on a scale from 1 (“not bad at all”) to 7 (“very bad”). Bonferroni-corrected pairwise comparisons between groups did not find any effect of instruction or questioning technique on perceived sensitivity of the question; participants in all experimental groups considered the topic under investigation to be equally sensitive.

For perceived confidentiality, both instruction and questioning technique exhibited significant main effects. Overall, participants in the “honest” condition reported higher levels of perceived confidentiality than participants in the “fake good” condition. Furthermore, perceived confidentiality was higher in the CWM than in the DQ condition. A significant interaction between instruction and questioning technique revealed that the advantage of the CWM over DQ was only observed among participants instructed to “fake good”; for participants instructed to respond “honestly”, the questioning technique did not affect perceived confidentiality.

With respect to the subjective ease of faking on the questionnaire, a significant main effect was found for questioning technique. Compared to participants in the DQ condition, participants in the CWM condition found it more difficult to respond in a way that did not make them appear to be a carrier of the sensitive attribute. This effect was found both in the “honest” and in the “fake good” conditions. Neither instruction nor the interaction between instruction and questioning technique affected the subjective ease of faking.

## Discussion

Dishonest responses due to deliberate faking and social desirability bias threaten the validity of survey results on sensitive attitudes and behaviors. Indirect questioning techniques such as the crosswise model [CWM; 19] promise to control social desirability bias by granting respondents higher confidentiality with respect to their individual answers. In a direct test of the CWM’s presumed ability to better control inflated self-presentations, we asked participants about a potentially sensitive behavior, whether they had crossed the street on a ‘Don’t Walk’ sign in plain sight of children within the past twelve months. Prevalence estimates for this socially undesirable behavior were generally higher and therefore presumably more valid in the CWM than in the DQ condition. Moreover, to directly evaluate the influence of deliberate faking, this study was the first to experimentally investigate the influence of “fake good” instructions on the validity of results from DQ versus CWM questions. In the DQ condition, less than half as many participants admitted to the sensitive behavior when instructed to “fake good” than when instructed to respond “honestly”, indicating that deliberate faking led to severe underestimation. In the CWM condition, however, prevalence estimates were unaffected by “honest” versus “fake good” instructions. Analyses of three additional measures of subjects’ experience of the survey showed that crossing the street on a ‘Don’t Walk’ sign in plain sight of children was indeed perceived as a sensitive behavior in all experimental groups; that CWM questions resulted in higher perceived confidentiality, though only under “fake good” instructions; and that participants in the CWM condition generally found it more difficult to fake their response in a way that would leave a positive impression.

Taken together, our results strongly suggest that direct self-reports of crossing the street on a ‘Don’t Walk’ sign in plain sight of children are influenced by social desirability bias, and that indirect questioning can help to obtain more valid estimates of the prevalence of this behavior. Estimates in the CWM condition were presumably less biased, as they exceeded those in the DQ condition, sufficing the “more is better”-criterion [56]. This finding is consistent with several other positive evaluations of the technique [e.g., 9,21,23,26,27,30].

More importantly, however, and as an important extension to the existing literature, CWM questions were shown to be much more robust against deliberate faking than DQ questions. In the DQ condition, “fake good” instructions heavily impaired the validity of estimates; participants were quite successful in faking their response in a way that made a more positive impression. In contrast, CWM estimates did not differ between the “honest” and “fake good” conditions. This finding is consistent with reports of higher perceived confidentiality and reduced ease of faking in CWM compared to DQ.

With respect to higher perceived confidentiality under “fake good” conditions, we argue that the CWM’s simple instructions are successful in helping respondents comprehend the rationale of the randomization process and how it protects the confidentiality of their answer. This interpretation is in line with a previous positive evaluation of the CWM’s comprehensibility and perceived privacy protection [39]. Especially when instructed to respond in a way that will leave a positive impression (that is, to “fake good”), respondents seem to understand that in the CWM, they can provide a truthful response to an embarrassing question without making a negative impression. Unexpectedly, however, participants instructed to respond honestly did not report higher perceived confidentiality when confronted with a CWM rather than with a conventional direct question. As a potential explanation for this finding, participants might have taken general components of the survey situation into account when evaluating perceived confidentiality, such as the anonymity of their participation or the minimal amount of personal information they had to report. These extraneous factors might have masked a potential effect of questioning techniques on perceived confidentiality. Conversely, the “fake good” instructions may have emphasized the sensitive nature of the behavior under investigation. They may thus have reminded participants that a direct question does not protect the confidentiality of their answers at all. Another potential explanation is that questioning technique was varied as a between-subjects factor in the current study. Participants in the DQ condition never saw a question in CWM format (and vice versa); hence, they were not able to establish a common frame of reference for these two conditions [cf. 72]. A within-subjects design would have presented participants with both question formats and would thus have allowed assessing the level of confidentiality they afford using the same frame of reference; this may have resulted in larger effect sizes. However, it was impossible to employ a within-subjects design in the current study, as directly answering the sensitive question first would have made a second presentation of the same question in an indirect format appear absurd. Future studies could combine a “fake good” manipulation with the scenario-based approach used in studies such as [39] to assess whether the two question formats differ in the perceived confidentiality they afford respondents.

As to the lower reported ease of faking, it seems that even if participants try to fake their answer to a CWM question, they cannot identify a self-protective response. We attribute this robustness towards deliberate faking to the symmetrical nature of the model [cf. 19]. As long as the prevalence of the sensitive attribute, the randomization probability, and the proportion of respondents choosing the first answer option are different from 0% and from 100% (all of which are true in the current study), there is no “safe” answer option respondents can choose to unambiguously deny being a carrier of the sensitive attribute. Moreover, it is impossible for respondents to assess the exact conditional probabilities of being identified as a carrier of the sensitive attribute when choosing either of the two answer options, as this would require knowledge of the prevalence of the sensitive attribute and the proportion of respondents choosing the first answer option. These values remain unknown to both the respondents and the experimenters until data collection is complete. Even if respondents tried to roughly assess the relative risk associated with either answer option, they would have to estimate the randomization probability *p* and derive and understand its effect on the conditional probability of being identified as a carrier when choosing either answer option. However, prior research has found that respondents are relatively poor at understanding the relationship between the randomization probability and the objective privacy protection in the context of indirect questioning techniques [43]. Against this background, we consider it highly unlikely that a substantial proportion of respondents will successfully make time-consuming inferences about the relative risk of the available answer options in short surveys. Hence, it seems that participants are doomed to fail when trying to “fake good” in the CWM.

The potential attribution of the robustness of the CWM to its response symmetry also relates to a first important limitation of our study. Due to limited resources, we decided to focus on the CWM because this model is associated with a comparatively high comprehensibility and perceived privacy protection, and an acceptable estimation efficiency [19,39]. These properties made it appear suitable for an initial investigation of the influence of “fake good” instructions on indirect questioning techniques. However, our comparison of CWM to DQ only allowed for the conclusion that the CWM is less susceptible to deliberate faking than direct questions. While the attribution of this robustness to the symmetry of the CWM seems highly plausible, this assumption needs to be tested explicitly in future studies that should also include asymmetric models such as, for example, the Triangular Model (TRM) [19]. In a recent comparison based on the “more is better”-criterion, the symmetric CWM has been shown to outperform the asymmetric TRM in terms of estimation validity [26]. For the proportion of respondents holding socially undesirable, Xenophobic attitudes, a higher and thus potentially more valid estimate was achieved in a CWM compared to a TRM condition. As CWM and TRM questions are very similar and only differ concerning the symmetry of their answer options, the superiority of the CWM was explicitly attributed to its response symmetry. In contrast, the availability of a “safe” answer option in the asymmetric TRM potentially allowed respondents to follow a self-protective answering strategy, thereby leading to an underestimation of the prevalence of Xenophobia. In light of these findings, we expect that when compared in terms of susceptibility to deliberate faking, the symmetric CWM will also yield more favorable results than the asymmetric TRM.

As a second limitation to our study, it should be noted that the “more is better” validation criterion allowed us to evaluate the validity of the prevalence estimates obtained only on a relative as opposed to an absolute level. Because the true prevalence of crossing the street on a ‘Don’t Walk’ sign in plain sight of children remained unknown, we cannot know whether any of the prevalence estimates we obtained in our sample closely reflected the true value, or whether they were still under-, or even overestimates. Even more importantly, the “more is better”-approach also did not allow testing for the influence of false positives or false negatives, as this would have required knowing the status of individual respondents concerning the sensitive attribute. False positives and false negatives in the CWM are likely a consequence of some respondents not understanding the comparatively complex instructions and therefore choosing one of the answer options at random. Especially in cases in which the true prevalence is zero or very low, false positives have been shown to lead to somewhat inflated CWM prevalence estimates [34,35]. In the current study, we cannot rule out that some participants in the CWM conditions disregarded the CWM instructions and chose their answers at random, potentially leading to some false positives. However, a recent study has shown that if the true prevalence of the sensitive attribute is well above 0% (as is likely the case in the current study), the influence of false negatives on CWM estimates outweighs the influence of false positives [38]. Therefore, even if random responding affected CWM estimates in the current study, overall, it most likely deflated rather than inflated CWM estimates and can therefore not explain the difference between DQ and CWM estimates. Most importantly, random responding cannot account for the interaction between instruction and questioning technique, reflected in the reduction of the large effect of faking instructions on respondents in the DQ condition to a close-to-zero effect on respondents confronted with a CWM question. It would be necessary to assume that virtually all respondents in the CWM conditions completely disregarded the “honest” versus “fake good” instructions and the instructions on how to operate the CWM question to explain this interaction solely based on random responding. This explanation appears highly unlikely given the observed effect of faking instructions in the DQ conditions, the random allocation to experimental conditions, and previous empirical studies suggesting false positive rates in the CWM of 5% to 14% rather than 100% [34,35,38]. Nevertheless, to counter the apparent limitations associated with the “more is better”-criterion, future studies should investigate the effect of deliberate faking on questions on sensitive attributes for which the true prevalence and the status of individual respondents are known or can be determined directly from the sample [cf. 30,34,38]. Only such strong validation studies can provide conclusive evidence for the absolute validity of the prevalence estimates obtained, on the absolute influence of false positives and false negatives due to random responding, and consequently on the absolute robustness of indirect questioning techniques such as the CWM against deliberate faking attempts.

A third limitation of our study is due to the method we used to elicit “honest” versus “fake good” responses. In the present first investigation of the influence of positive self-presentation on estimates obtained via indirect questioning, we opted for an experimental manipulation of respondent honesty via “fake good” instructions. We preferred this approach over a non-experimental comparison of real-world situations providing more serious and potentially differing incentives to fake, for example, in a study of job applicants. This decision was taken to maximize internal validity even though this choice was associated with limitations in terms of the ecological validity of our study. Our design allowed us to attribute observed group differences to our experimental manipulation of respondent honesty. In contrast, a non-experimental design would have been open to potential alternative explanations, including, for example, confounding variables or self-selection effects. Presumably, because of the high degree of experimental control they afford, similar experimental “fake good” manipulations have been - and still are - successfully applied to investigate the influence of positive self-presentation on various measures of self-report [49–55]. While the CWM has proven to be robust against an experimenter-instructed “faking good” in the current study, the generalizability of this finding should be tested further due to the peculiar nature of this manipulation. Future studies need to evaluate whether the robustness of the model is also maintained in real-world situations presenting high incentives towards deliberate, positive self-presentation.

A fourth limitation of the current study is the composition of our sample, which exclusively comprised people with a high level of education, that is, university students. As educational attainment and academic performance have repeatedly been shown to be positively, and strongly, associated with cognitive ability [73–75], the generalizability of our results is also potentially restricted to people with comparatively high cognitive abilities. In less educated samples, indirect questioning techniques have been found to be associated with lower acceptance rates [76], lower comprehensibility [39], and a higher share of participants disregarding the instructions [77]. Hence, future studies should include respondents’ level of education as a quasi-experimental factor to investigate its effect on the ability to deliberately fake on direct versus indirect questions. In such a design, a recently proposed extension of the crosswise model, the ECWM, might additionally be employed to quantify the share of participants who do not follow the instructions [78,79].

Finally, we would like to encourage researchers to contrast indirect questioning techniques such as the CWM with alternative approaches to measuring and controlling the influence of deliberate faking on self-reports such as, for example, social desirability scales [3,80,81], behavioral indicators [82–84], lie detection and the bogus pipeline [85–87], and the overclaiming technique [88–90]. Such extended studies could help identify those methods–or possibly even a combination of methods–that optimally counteract the detrimental influence of deliberate positive self-presentation on the validity of self-reports.

## Conclusion

In summary, we have demonstrated that the crosswise model is capable of controlling for the influence of deliberate faking in surveys on sensitive issues. This robustness of the CWM is presumably attributable to the higher level of perceived confidentiality it affords compared to conventional direct questions, and to respondents’ inability to fake their answers in a self-protective manner in the CWM question format. We therefore recommend using the CWM in surveys on sensitive personal attitudes and behaviors to reduce bias due to self-presentational concerns and minimize the influence of deliberate faking.
